# Research trends in Rift Valley fever virus: a bibliometric analysis from 1936 to 2024

**DOI:** 10.3389/fvets.2025.1611642

**Published:** 2025-07-09

**Authors:** Huiying Zhang, Shuai Song, Leiliang Zhang

**Affiliations:** ^1^Department of Clinical Laboratory Medicine, The First Affiliated Hospital of Shandong First Medical University and Shandong Provincial Qianfoshan Hospital, Jinan, Shandong, China; ^2^Department of Pathogen Biology, School of Clinical and Basic Medical Sciences, Shandong First Medical University and Shandong Academy of Medical Sciences, Jinan, Shandong, China

**Keywords:** RVF, RVFV, bibliometric, CiteSpace, VOSviewer

## Abstract

**Background:**

Rift Valley fever (RVF), first identified in Kenya in 1930, is a viral zoonosis transmitted by arthropods and poses significant risks to both public and animal health. Despite the wealth of published studies on the disease, the number of bibliometric analysis of Rift Valley fever virus (RVFV) is limited. This study utilized bibliometric analysis to identify research hotspots and emerging trends in RVFV studies, aiming to offer new insights and strategic references for future research directions and prevention strategies.

**Methods:**

The study utilized Scopus to collect global publications on RVFV from 1936 to 2024. Data processing and visualization were performed with VOSviewer, CiteSpace, Scimago Graphica, and the Bibliometrix web tool.

**Results:**

A total of 1,629 publications related to RVFV were included in this analysis. Geographic distribution showed that the United States, France, and Kenya were the most productive countries in this field, while Institut Pasteur emerged as the leading research institution. PLoS Neglected Tropical Diseases was identified as the predominant journal for publishing RVFV research, while Virology received the highest citation frequency. Author analysis revealed Clarence Peters as the most prolific contributor, with “Rift Valley fever virus” being the most frequently used keyword.

**Conclusion:**

This bibliometric investigation systematically assessed nine decades of research on RVFV, providing a comprehensive overview of the contributions from leading nations, institutions, researchers, and journals in this field. Global interest in RVFV research has been steadily increasing, particularly in recent years. This surge in attention, largely influenced by climate change, has attracted heightened focus from public health departments.

## 1 Introduction

Rift Valley fever (RVF) is a mosquito-borne viral zoonosis, first detected in Kenya in 1930 and now endemic in several African countries and the Arabian Peninsula, with an ecologically complex transmission mechanism that requires a combination of environmental elements and biological vectors involving mosquitoes, humans, and livestock (cows, sheep, goats, camels, and buffaloes) (1, 2). Rift Valley fever virus (RVFV), the etiological agent of RVF, is classified within the genus *Phlebovirus* in family *Phenuiviridae*.

The virus perpetuates in enzootic cycles via mosquito vectors among domestic ruminants, while human infection occurs predominantly through direct contact with infected animal materials. Notably, epidemiological evidence confirms the absence of interhuman transmission (3, 4). Human infections can lead to a wide range of clinical outcomes, with the majority of infected individuals presenting with self-resolving febrile symptoms and approximately 1–2% of cases progressing to severe disease with high mortality (5). RVF has diverse clinical manifestations. Mild cases usually present with flu-like symptoms, including persistent fever, severe headache, low back muscle pain, marked dizziness, loss of appetite and photophobia (6). Severe cases may involve multiple organ systems, mainly in the form of: (1) hepatitis, jaundice, and bleeding disorders (7); (2) encephalitis and neurologic disorders (8); (3) encompass a range of conditions, including macular and parafoveal edema, uveitis, retinitis, and retinal hemorrhages (9); and (4) miscarriages (10). Viral neutralization tests with high sensitivity and specificity are the gold standard for detecting past RVFV exposures but have the disadvantage of having to be performed in a laboratory setting where live RVFV can be safely handled (11). Research on the prevention and control of RVF has shown a multidisciplinary development involving vaccines, specific antiviral drugs, optimization of public health strategies, etc., but a comprehensive prevention and control system has yet to be formed. The development of veterinary vaccines against RVF has achieved a breakthrough. Still, different testing and evaluation stages are required, and the development of preventive vaccines for human beings urgently needs to strengthen basic research and clinical exploration. Currently, there are no approved effective drugs for the clinical treatment of RVF, and the treatment is mainly symptomatic and supportive, with a variety of antiviral drugs and antibody therapies still in the preclinical stage. RVF continues to threaten livestock and human health, posing a major public health risk. The World Health Organization (WHO) has classified it as a priority infectious disease for research and development and included it in the list of diseases requiring accelerated research and development in emergency situations (12).

Bibliometrics assesses research trends, disciplinary development, influence of institutions and scholars, and knowledge structure through quantitative analysis of academic literature. By comprehensively examining multidimensional indicators such as countries, institutions, scholars, journals, and subject headings, bibliometrics quantitatively characterizes the current status of scientific research and predicts its development. This approach enables an objective representation of disciplinary knowledge structures and detection of research hotspots. While research on RVFV has expanded significantly in recent years, systematic bibliometric analyses in this field remain comparatively limited. This study introduces several methodological improvements compared to previous seroprevalence research (13). First, it innovatively incorporates the framework of bibliometric visualization analysis technology. Second, it updates the database selection process. Third, the study applies more diverse and advanced analysis software, greatly enhancing the presentation dimensions of the visualization map. New visualization modules, such as the Keyword Timezone Map and the Keyword Outbreak Map, have been added, allowing for a clear depiction of the evolution of research hotspots. Notably, this study includes a dedicated section analyzing references, providing broader coverage of the knowledge base in current research and facilitating a more comprehensive and in-depth exploration of the disciplinary knowledge framework.

This study systematically compiled the research hotspots and cutting-edge directions in this field by mapping the scientific knowledge system and comprehensively analyzing the RVFV research literature over the past 90 years. This provides an important theoretical basis and data support for future in-depth research on RVFV and the development of prevention and treatment strategies.

## 2 Materials and methods

### 2.1 Data sources and search strategy

**Figure 1:**
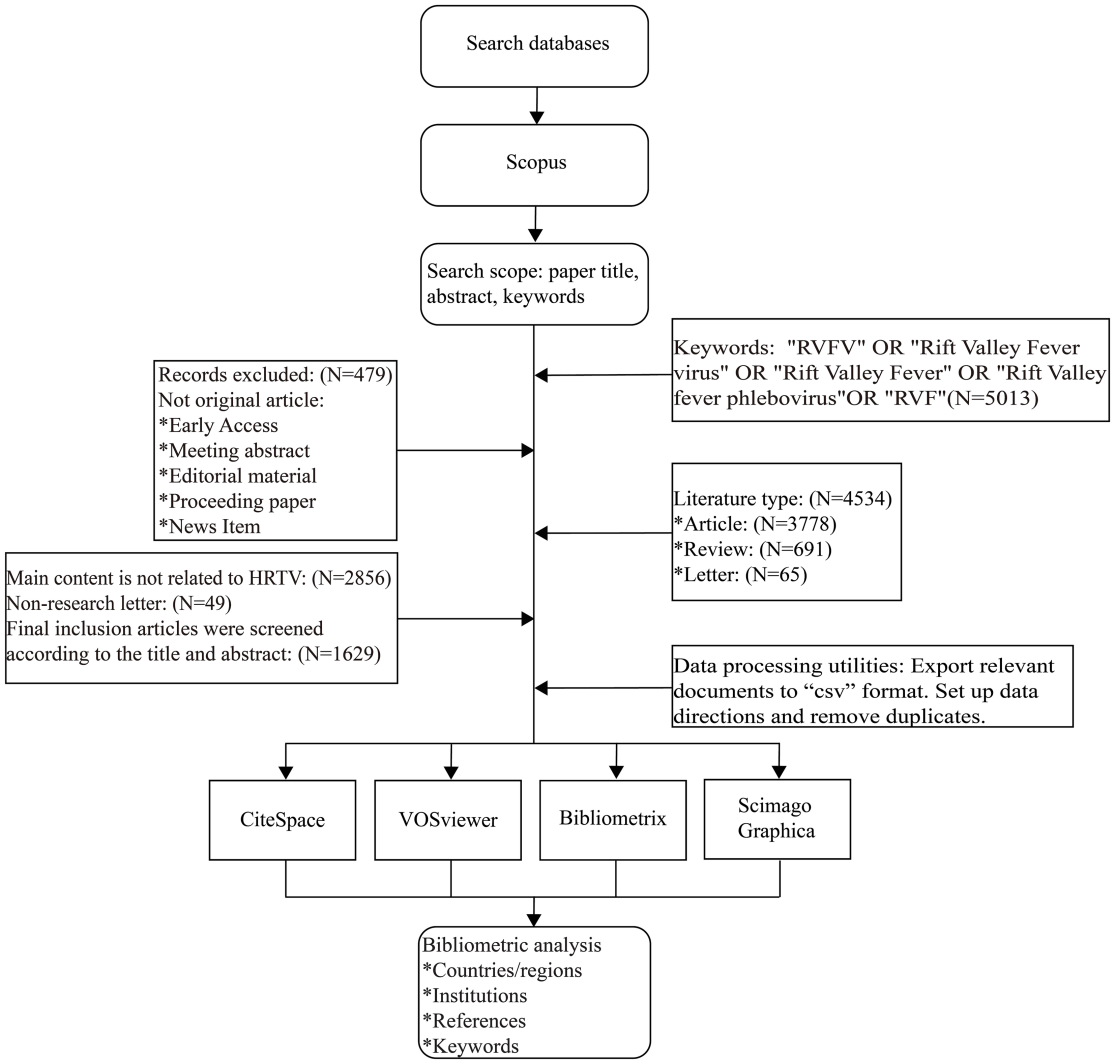
Process of data retrieval.

This study utilizes the Scopus database as the core data source. As a leading global platform for literature retrieval and analysis, Scopus demonstrates exceptional value in integrating academic resources and evaluating scientific research, making it a preferred tool for scholarly literature investigations. Literature was retrieved from Scopus using these search terms: Topic = (“RVFV” OR “Rift Valley fever virus” OR “Rift Valley fever” OR “Rift Valley fever phlebovirus” OR “RVF”). This study searched the Scopus database for all literature published from January 1, 1936, to October 25, 2024, in the Scopus database. The search criteria included: (1) literature types such as articles, reviews, and research letters published in English, and (2) content directly related to RVFV. Two independent researchers synchronized the literature search and screening process to ensure data timeliness and accuracy. The search strategy was based on a core screening of titles, abstracts, and keywords, and a full-text review of potentially relevant literature was conducted to ensure its fit with the study topic. During the literature screening phase, particular attention should be given to publications containing significant citation data. The specific screening procedure comprises: (1) exporting CSV format datasets of the selected literature from the Scopus database; (2) converting the data into TXT format text files through CiteSpace analysis software to meet the requirements of subsequent research and analysis. The complete technology roadmap is detailed in Figure 1. Since all data in this study were obtained from public databases, no additional approval from the Ethics Committee was required.

### 2.2 Data analysis and visualization

The core strength of bibliometrics lies in its data-driven objectivity and trend prediction ability. It can identify disciplinary evolution and emerging areas through keyword co-occurrence, citation networks, etc., and its reliability has been further strengthened by the widespread use of databases such as Scopus and the Web of Science. In this study, four professional analysis tools were comprehensively used in the knowledge graph construction process: CiteSpace (version 6.4. R1), VOSviewer (version 1.6.20), Scimago Graphica (version 1.0.35.0), and Bibliometrix online analysis platform. Each of these tools has its own characteristics and complementary functions, significantly improving the efficiency of knowledge graph construction and in-depth analysis capabilities. Among them, CiteSpace, a visual analysis platform based on the principle of set theory, has its core advantage in data standardization processing. By integrating clustering algorithms and knowledge unit similarity metrics, the software systematically reveals the development trajectory and evolutionary trends of the RVFV research field, thus providing a scientific basis for the study of cutting-edge dynamics in the discipline (14). VOSviewer, through core functions such as keyword co-occurrence analysis, institutional collaboration networks, and author co-occurrence networks, can visually present the development trends, collaboration characteristics, and related association dimensions in the research field of RVFV. VOSviewer can provide three visualization patterns: The network visualization exhibits the overall correlation structure, the overlay visualization reflects the characteristics of temporal evolution, and the density visualization highlights the distribution of research hotspots, offering a systematic solution for the multi-dimensional presentation of scientific research data (14).

In this study, the data cleaning function of CiteSpace was used to systematically de-duplicate the Scopus literature dataset in the data preprocessing process. We have established a rigorous and standardized process for all documentation to ensure data quality. Specific operations include: (1) Utilizing VOSviewer’s term management system to create standard text files according to the “thesaurus_authors” specification; (2) Performing term consolidation in this TXT format file, which involves: (a) consolidating synonyms, (b) grouping related terms into higher-level categories, and (c) standardizing regional administrative units to the national level. Once preprocessing is complete, the thesaurus file is imported into the cluster analysis interface of VOSviewer to achieve normalized data deduplication. This approach is well-suited for data integration across countries, institutions, and references.

## 3 Results

### 3.1 Trends of publications

**Figure 2:**
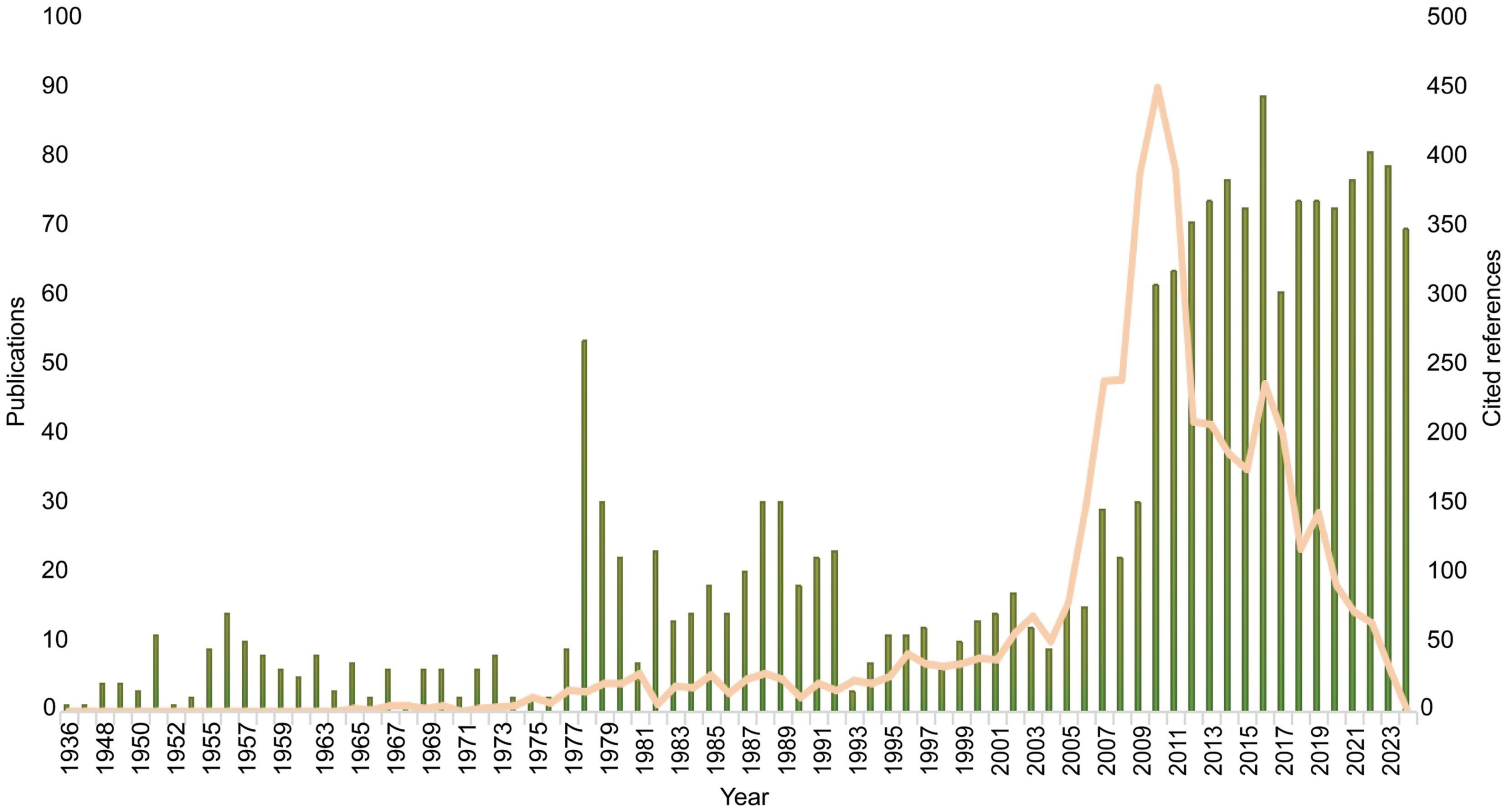
Trends in the number of publications and major cited references from 1936 to 2024.

CiteSpace software was used to process the search results derived from the Scopus database. A total of 1,629 documents meeting the search criteria were selected in this study. As shown in Figure 2, among the literature included in this study, the number of published papers in the RVFV field exhibited two distinct peaks in 1978 and 2016, surged in 2010, and has maintained a consistently high output since then. Specifically, the annual number of publications maintained a steady growth and the cited frequency of references increased year by year, among which the annual number of cited references showed a significant growth peak from 2005 to 2019, which fully confirmed that RVFV research continued to receive close attention in the academic community. Although important breakthroughs have been made in basic virology research and prevention and control technologies, significant research gaps remain in key areas such as virus pathogenesis, host interaction mechanism, novel vaccine research and development, and specific antiviral therapy.

### 3.2 Contributions of countries/regions

Academic contributions to RVFV research have come from 154 countries/regions, with the top five publishing nations detailed in Table 1. Statistics show that the United States topped the list with 620 articles, followed by France (*n* = 190) and Kenya (*n* = 153). Total Link Strength (TLS) directly reflects the frequency of cooperation between countries, and Centrality represents the hub status of a country in the research international cooperation network. These two key indicators are crucial for analyzing data on international research cooperation. The United States ranks first in both indicators, highlighting its central position and academic leadership in international cooperation in this RVFV area.

**Table 1:** Top 5 countries with the highest number of publications in the RVFV field in this study.

Rank	Country	Publications	Total citations	Average citations	Total link strength	Centrality
1	United States	620	24,565	39.62	376	0.37
2	France	190	7,656	40.29	237	0.23
3	Kenya	153	5,662	37.01	212	0.17
4	South Africa	145	5,296	36.52	174	0.06
5	United Kingdom	131	4,042	20.85	187	0.12

**Figure 3:**
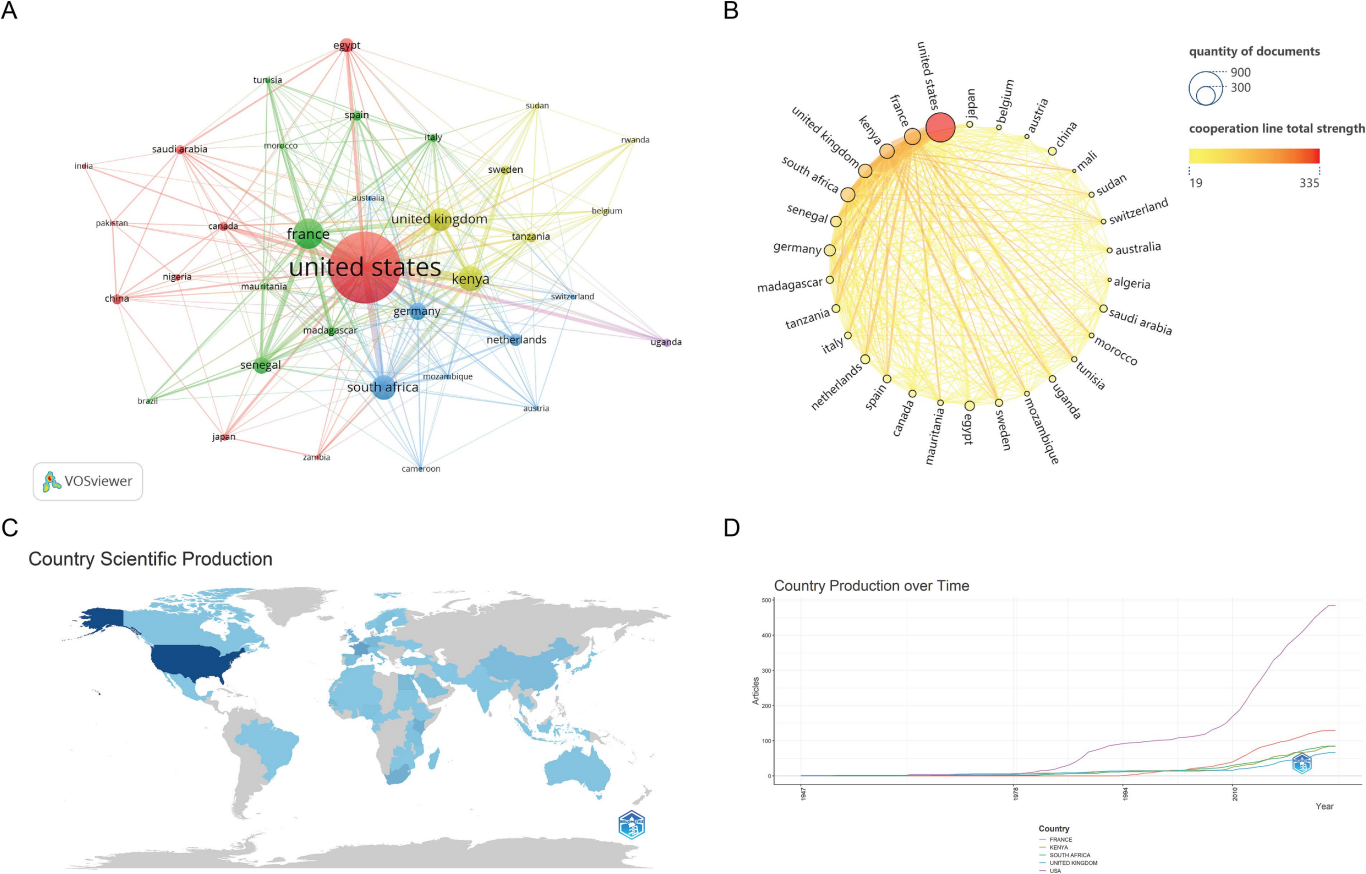
Visualization and analysis of international cooperation networks in the RVFV field in this study. **(A)** Cooperation clustering map of countries. **(B)** Map of the number of publications and cooperation intensity of countries. **(C)** Map of scientific production of countries. **(D)** The trend chart of the scientific research output of the top five countries with the number of published documents over time.

In this study, VOSviewer was used to visually analyze the scientific research publications of countries and regions in the RVFV research field, and at least 10 publications were set as the inclusion criteria. The final 35 countries and regions that met the requirements are shown in Figure 3A. In the network, node size indicates national publication volume and link thickness shows cooperation intensity between countries. Figure 3B presents the Scimago Graphica-generated visualization of national research productivity and collaborative networks, simultaneously displaying the distribution patterns of research output magnitude and international cooperation density. The size of the circle in the figure is proportional to the number of publications, and the larger the circle, the higher the number of publications in a country. The depth of the color of the circle and the line and the thickness of the line is proportional to the total intensity of the cooperation, and the darker the color and the thicker the line indicates, the more prominent the country’s contribution to international cooperation—Figures 3C,D visualize national scientific output and publication trends of top 5 countries through Bibliometrix. Among them, the color depth in Figure 3C is positively correlated with the scientific research output; Figure 3D shows that the output growth trend of the leading countries in the RVFV field was relatively flat before 2010 and then accelerated significantly after 2010, indicating that this period was an important breakthrough stage in RVFV research. The RVF epidemic experienced a significant outbreak and severe cross-border spread in the African region, particularly centered on Kenya, from 2006 to 2007, posing an imminent health threat to Europe, Asia, and the Americas from 2006 to 2010.

### 3.3 Analysis of institutions

**Figure 4:**
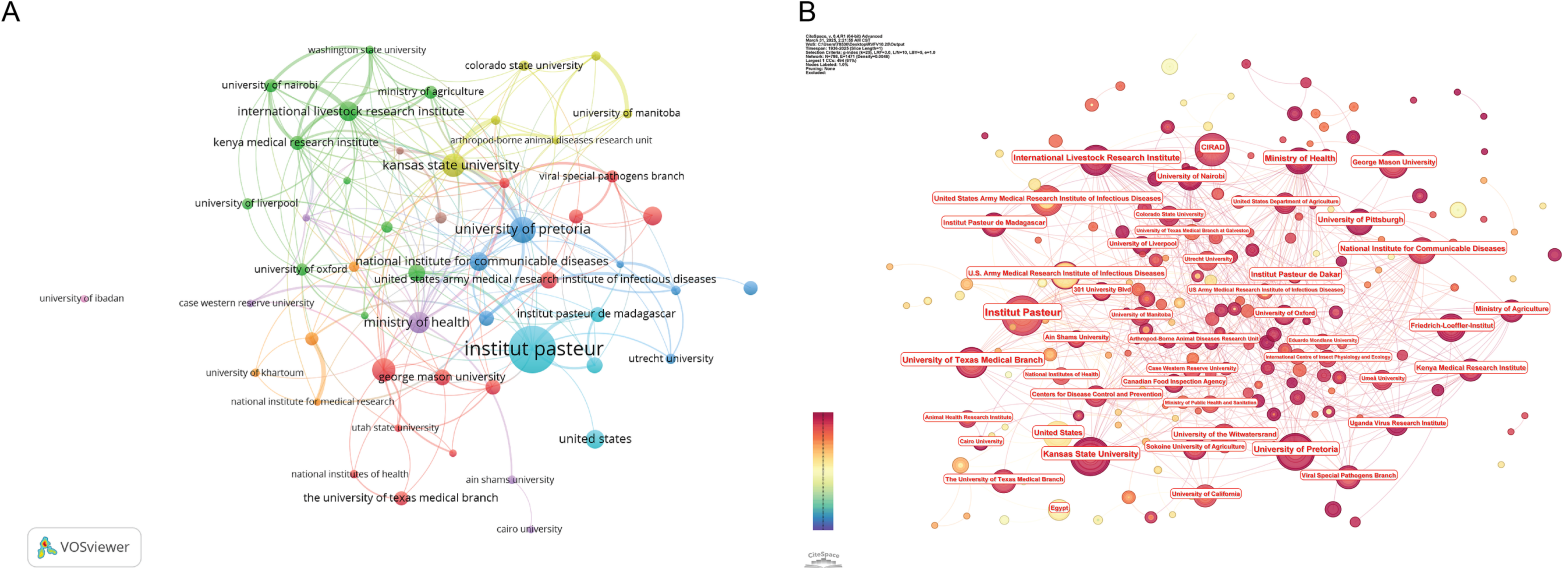
Visualization and analysis of research institutions in the RVFV field in this study. **(A)** Cooperation map of 52 institutions with the number of publications no less than 10. **(B)** The visualization map of the number and time of publication of institutions.

According to statistics, a total of 1964 research institutions worldwide have participated in RVFV-related research and published academic articles (Table 2). Among them, the number of French scientific research institutions is significantly ahead of other countries, highlighting France’s important position in the field of research. Specifically, the Institut Pasteur topped the list with 104 publications, followed by the University of Pretoria (*n* = 50) and Kansas State University (*n* = 43). A total of 52 eligible institutions were selected through VOSviewer software analysis (setting the minimum number of publications to 10) (Figure 4A). In this visual network, the size of nodes is proportional to the number of publications, and the thickness of connection lines reflects the intensity of inter-institutional cooperation. The results of the CiteSpace analysis (Figure 4B) further illustrate the trend of publications by each institution over time using a color gradient, with color shifting from light to dark to indicate the timeline from earlier to more recent publications. Combining the results of the analysis in Figures 4A,B, the Institut Pasteur was recognized as the most influential research institution in the field of RVFV research. Institutions with high research output are usually leaders in the field, and their research direction may represent the frontier of the discipline. Interinstitutional co-occurrence analysis can reveal the mode and intensity of cooperation between different institutions, which helps to find potential cross-institutional and transnational cooperation opportunities and promote the sharing of scientific research resources.

**Table 2:** Top five institutions with the highest number of publications in the RVFV field in this study.

Rank	Institution	Publications	Citations	Countries	Average citation/publication	Total link strength
1	Institut Pasteur	104	6,146	France	59.07	50
2	University of Pretoria	50	1,134	South Africa	22.68	69
3	Kansas State University	43	780	United States	18.14	35
4	University of Texas Medical Branch	43	1841	United States	42.81	34
5	Ministry of Health	39	1946	Kenya	49.90	61

### 3.4 Analysis of authors

**Figure 5:**
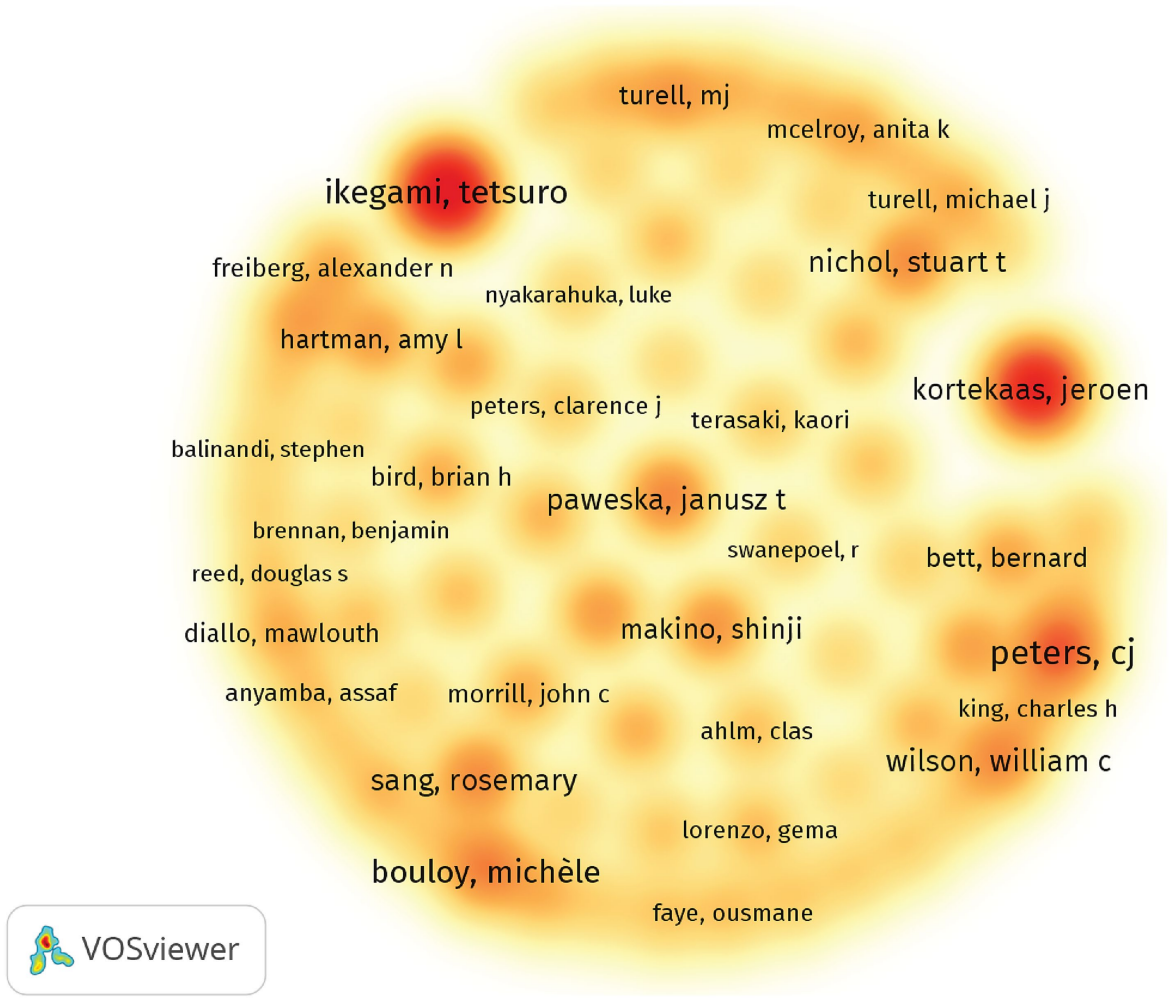
Density visualization of authors by the number of publications.

The top three authors based on the number of publications are C. J. Peters (*n* = 54), Tetsuro Ikegami (*n* = 47), and Michèle Bouloy (*n* = 38) (Table 3). Using VOSviewer software for author density visualization by country/region, high-density areas typically indicate countries where authors are more active in this field, while low-density areas suggest either less research activity or insufficient attention to the field. Figure 5 illustrates that authors from countries such as the United States and France have made significant contributions to the research on RVF. These two countries appear to hold a leading position in the academic landscape of RVFV research, owing to their substantial research funding, well-established infrastructure, and active international collaboration networks. This situation highlights the existing cooperation gaps between these European and American nations and African countries, particularly those like Kenya, which face higher local epidemic pressures. Strengthening international collaboration, closing these gaps, and enhancing local scientific research capabilities in Africa would greatly benefit the advancement of research in this critical area.

**Table 3:** Top 10 cited-references in the RVFV field in this study.

Rank	Author	Citations	Year	Journals	DOI
1	Ikegami, Tetsuro	164	2011	Viruses	10.3390/v3050493
2	Linthicum, Kenneth J	117	1999	Science	10.1126/science.285.5426.397
3	Madani, TA	92	2003	Clin Infect Dis	10.1086/378747
4	Daubney, R	91	1931	Journal of Pathology and Bacteriology	10.1002/path.1700340418
5	Bird, BH	82	2009	Journal of the American Veterinary Medical Association	10.2460/javma.234.7.883
6	Dungu, B	78	2010	Vaccine	10.1016/j.vaccine.2010.04.085
7	Le May, N	73	2004	Cell	10.1016/S0092-8674(04)00132-1
8	Pepin, M	73	2010	Veterinary Research	10.1051/vetres/2010033
9	Muller, R	71	1995	American Journal of Tropical Medicine and Hygiene	10.4269/ajtmh.1995.53.405
10	Morrill, J C	69	2010	Vaccine	10.1016/0264-410X(91)90314-V

### 3.5 Analysis of references

**Figure 6:**
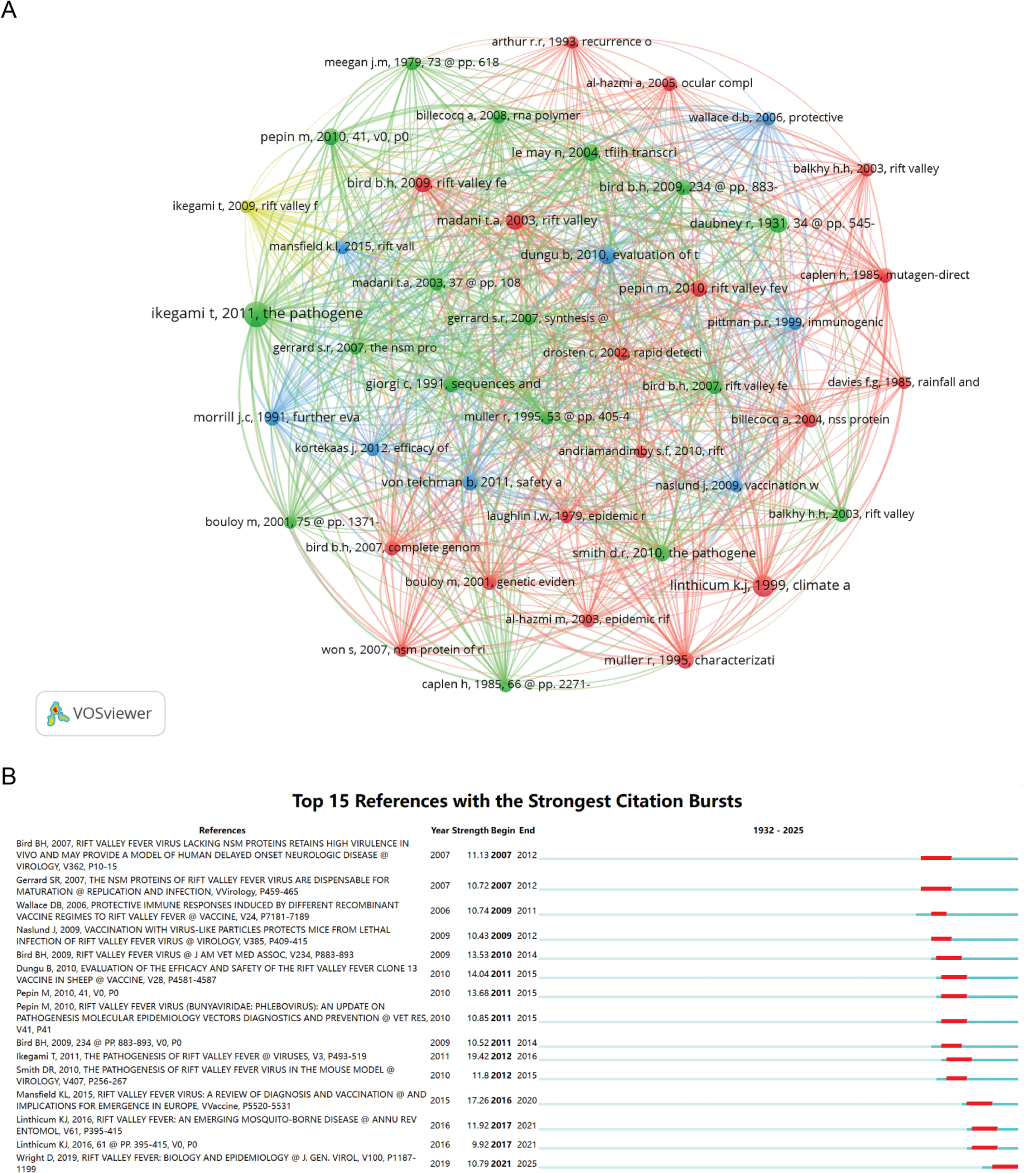
Visualization and analysis of the references in the RVFV field in this study. **(A)** Network map of references. **(B)** Top 15 references with the strongest citation bursts.

The 1,629 analyzed articles collectively referenced 34,908 citations. As shown in Table 4, Tetsuro Ikegami’s work emerged as the most frequently co-cited publication, indicating its significant scholarly impact on RVFV research. VOSviewer’s citation analysis revealed distinct and statistically significant clustering patterns within the co-citation network. The minimum number of references required was 40, with 44 articles meeting this threshold (Figure 6A). All cited literature was divided into four clusters, with node colors representing different clusters, node size and word size reflecting citation frequency, and connections illustrating co-occurrence. Figure 6B displays the top 15 references with the most pronounced citation bursts, highlighting shifts in RVFV research hotspots over time, with the red portions indicating periods of heightened relevance. Notably, Tetsuro Ikegami’s article, “The Pathogenesis of Rift Valley Fever,” published in 2011, was listed as one of the most frequently cited works in the literature included in our analysis. With a citation intensity of 19.42, this seminal paper stands out as the most cited in our analysis, underscoring its foundational contribution to RVFV research.

**Table 4:** Top 10 journals with the highest number of publications and citations in the RVFV field in this study.

Rank	Publication journal	Documents	Citations	IF*	Cited journal	Co-citations	IF*
1	PLoS Neglected Tropical Diseases	110	2,989	3.4	Virology	437	2.8
2	American Journal of Tropical Medicine and Hygiene	72	3,721	1.9	Vaccine	358	4.5
3	Journal of Virology	70	4,737	4.0	American Journal of Tropical Medicine and Hygiene	301	1.9
4	PLoS One	49	1,232	2.9	Emerging Infectious Diseases	273	7.2
5	Viruses	46	626	3.8	Viruses	259	3.8
6	Emerging Infectious Diseases	41	2,150	7.2	Plos One	254	2.9
7	Vaccine	40	1990	4.5	Diagnostics	240	3.0
8	Virology	40	2,112	2.8	Journal of Virology	238	4.0
9	Vector-Borne and Zoonotic Diseases	36	965	1.8	Science	226	44.8
10	Journal of Virological Methods	29	977	2.2	Lancet	192	98.4

IF*: impact factor based on the 2023 data from Web of Science.

### 3.6 Analysis of journals

The 1,629 publications included in this study appeared in 327 distinct journals, with 33 journals contributing 10 or more articles each. As shown in Table 4, the top 10 most productive journals in RVFV research are highlighted. The top three journals were PLoS Neglected Tropical Diseases (IF = 3.4), American Journal of Tropical Medicine and Hygiene (IF = 1.9), and Journal of Virology (IF = 4.0), which published 110, 72, and 70 articles, respectively. In terms of citations, Virology (IF = 2.8), Vaccine (IF = 4.5), and the American Journal of Tropical Medicine (IF = 1.9) ranked as the top three cited journals, with 437, 358, and 301 co-citations, respectively. It should be noted that the impact factors of the above journals are all from the 2023 data of Web of Science.

**Figure 7:**
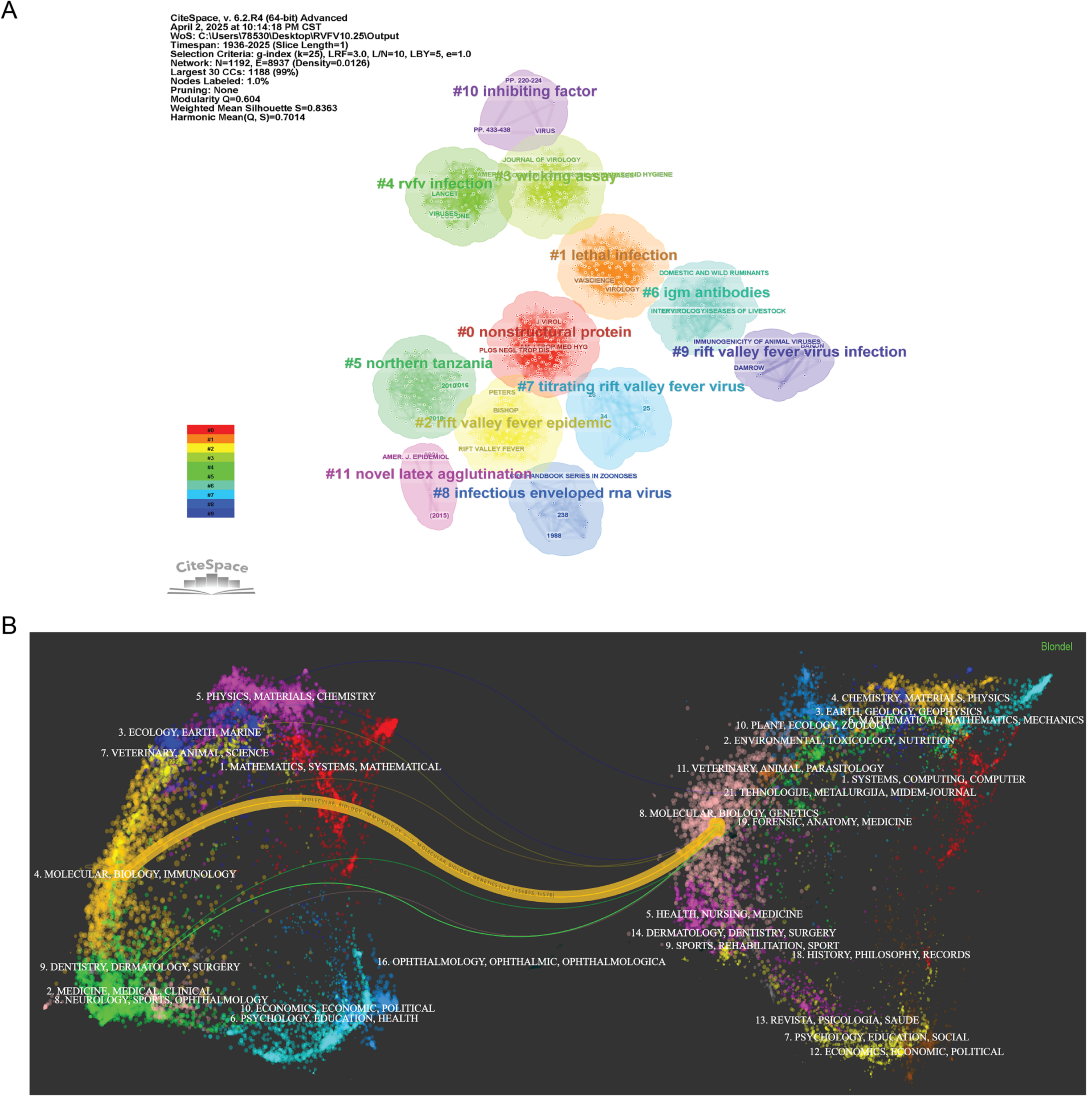
Visualization and analysis of the top journals in the RVFV field in this study. **(A)** Journal clustering network. **(B)** Dual-map of journals on RVF research.

Figure 7A presents the journal clustering analysis conducted using the CiteSpace journal clustering function, where “Title words” were used to label clusters. This analysis identified 12 significant clusters related to RVFV publications, including #nonstructural protein, #lethal infection, #Rift Valley fever epidemic, #wicking assay, #RVFV infection, #northern Tanzania, #IgM antibodies, #titrating Rift Valley fever virus, #infectious enveloped RNA virus, #Rift Valley fever virus infection, #inhibiting factor, and #novel latex agglutination. The clustering analysis yielded strong quality metrics, with a Modularity Q value of 0.604 (exceeding the 0.3 threshold) and a weighted mean Silhouette S score of 0.8363 (surpassing the 0.7 benchmark). These robust values confirm that the co-citation network displays statistically significant and conceptually meaningful clustering structures. The high Modularity Q score and weighted mean Silhouette S value indicate optimal clustering performance and strong network coherence.

Figure 7B presents the journal dual-map overlay of RVFV research generated through CiteSpace analysis. The clusters on the left represent citing journals, while those on the right represent cited journals. The dual map of journals reveals two primary paths for the citing and cited journals: (1) Molecular Biology and Immunology—Molecular Biology and Genetics; and (2) Medicine, Medical, Clinical—Molecular Biology and Genetics. The cited journals predominantly fall within the categories of Molecular Biology and Genetics.

### 3.7 Analysis of keywords

**Figure 8:**
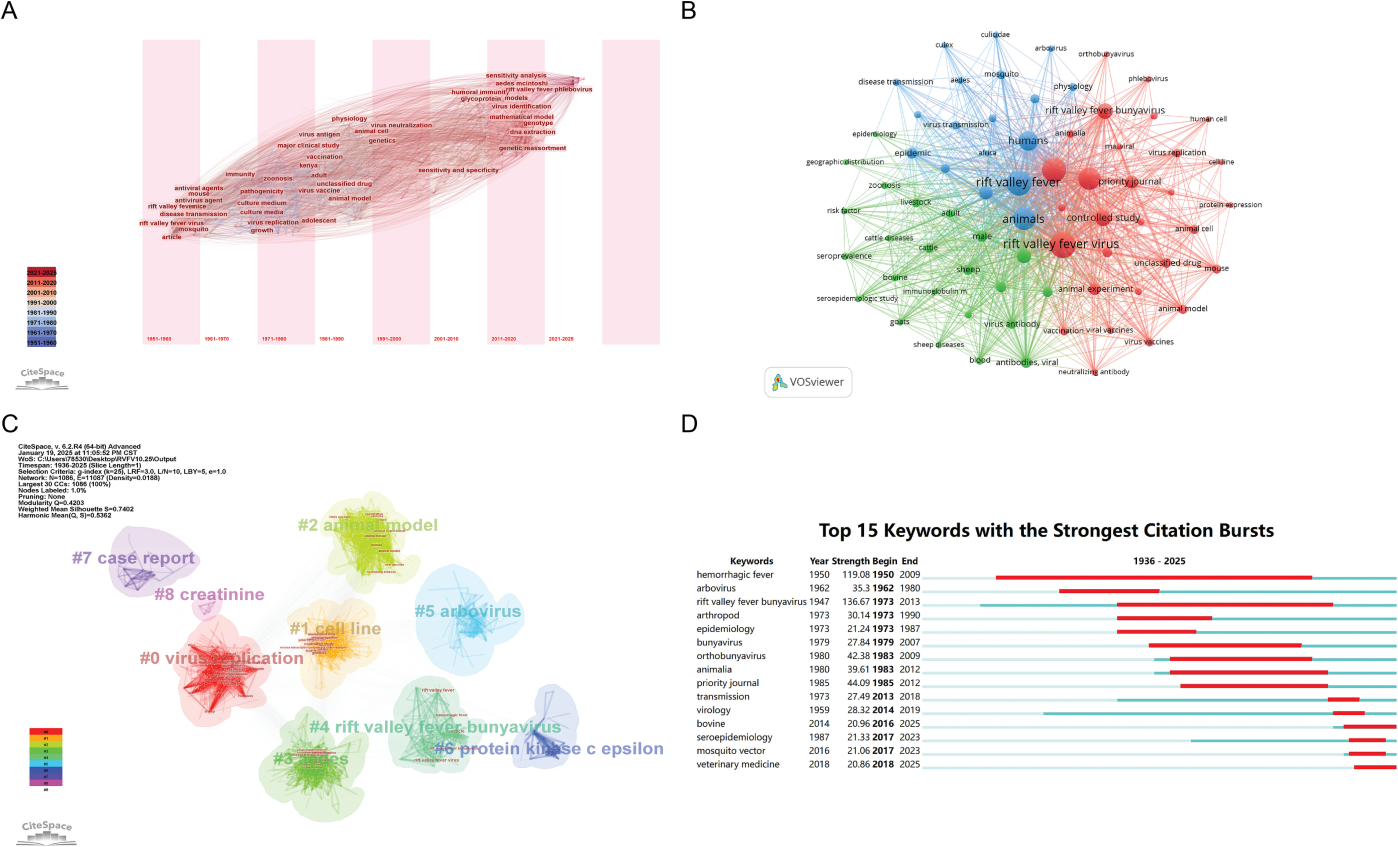
Visualization and analysis of the keywords in the RVFV field in this study. **(A)** Time zone map of keywords. **(B)** Network map of keywords. **(C)** Keywords clustering network. **(D)** Top 15 keywords with the strongest citation bursts.

Keyword analysis is a crucial component of bibliometric research as it helps to reveal the core themes developmental trends and research hotspots in RVFV studies. The most frequent keyword listed in Table 5 is “Rift Valley fever virus” with 1,261 occurrences in the literature. Figure 8A Illustrates the evolution and migration of keywords over time. Before 1970 the primary keywords concerning the etiology and transmission mechanisms of RVFV included “mosquito” “disease transmission” and “virus.” the period from 1970 to 2000 was marked by clinical research and vaccine development with keywords such as “virus vaccine” “major clinical study” and “animal model” emerging during this time. Since 2000 research has shifted toward understanding molecular mechanisms and precise identification of viruses with main keywords including “genetic reassortment”“virus identification” and “humoral immunity” reflecting the distribution of keywords across different periods.

**Table 5:** Top 10 keywords with the highest frequencies in the RVFV field in this study.

Rank	Keywords	Frequency
1	Rift Valley fever virus	1,261
2	Rift Valley fever	1,250
3	Animals	898
4	Controlled study	509
5	Virology	388
6	Priority journal	384
7	Animal experiment	314
8	Antibodies, viral	296
9	Epidemic	280
10	Enzyme linked immunosorbent assay	257

Keywords can be summarized into three categories and divided into nine clusters with different colors representing distinct clusters (Figures 8B,C). “Virus replication” “protein kinase C epsilon” “cell line” and “animal model” are classified under basic science research. This indicates that the focus is on understanding the mechanisms of viral replication (such as the inhibition of the host interferon pathway by the NSs protein) and the use of Vero and BHK-21 cell lines as well as animal models to study the pathogenicity of the virus. The keywords “Aedes” “vaccination” and “arbovirus” fall within the category of epidemiology and control techniques highlighting an interdisciplinary integration of mosquito-borne ecology viral transmission dynamics and vaccination coverage studies. Lastly “case report” and “Rift Valley fever” pertain to clinical cases and diagnoses indicating a synthesis of experiences in treating severe cases (e.g., hemorrhagic fever retinitis) to address the ongoing threat posed by this zoonotic disease

The top 15 keywords with the strongest citation bursts are shown in Figure 8D. Notably, the most frequently cited keyword during outbreaks is “Rift Valley fever bunyavirus” (intensity = 136.67), followed by “hemorrhagic fever” (119.08) and “priority journal” (44.09). As a zoonotic disease, RVFV outbreaks in animal populations often precede human cases, with veterinarians playing a key role in monitoring, controlling, and preventing RVFV. For instance, animal vaccines developed by the veterinary field are essential tools for combating RVFV. Consequently, “veterinarian” has emerged as a prominent topic in recent keyword outbreaks.

## 4 Discussion

RVF was first identified in 1931 during an epidemiological investigation of sheep flocks in Kenya’s Rift Valley region, from which the disease derives its name. The limited early literature on RVF reflects its recent discovery and the scarcity of foundational research at that time (2, 15). The RVF outbreak in Kenya during 1950–1951 saw a slight increase in publications compared to the 1930s (16), and the number of publications began to rise more significantly in the late 1970s to early 1980s, closely related to the serious RVF outbreak in Egypt during 1977–1978 (17). The RVF outbreak in Mauritania, West Africa, in 1987 significantly impacted international security (18), which may have echoed the outbreaks in the region as reflected in increased publications during the latter half of the 1980s. In the late 1990s, a severe RVF outbreak occurred in Kenya and Somalia, causing widespread concern in East Africa and increasing international investment in research on the disease (19). This was reflected by a publication trend that rose from the late 1990s to early 2000s. The first large-scale outbreaks of RVF outside Africa occurred in Saudi Arabia and Yemen in September 2000, raising concerns in non-endemic regions and prompting further research on RVF (20). By 2010, RVF outbreaks were reported multiple times in South Africa (21, 22). The number of publications has remained high since 2009, with a large number of collaborative studies and high-impact papers illustrating the surge in research and attention toward RVF as an acute infectious disease and as a wake-up call for global public health. The focus on RVF prevention and control has gradually shifted from being a regional issue to a global health security challenge.

Bibliometric analysis provides valuable insights into the evolution of research topics. For instance, by examining the time-zone distribution map of keywords, we can trace changes in research focus over time. The intensity, along with the start and end years reflected in the keyword outbreak map, highlights key research trends and timelines necessary for understanding the evolving areas of interest. For example, the co-occurrence intensity of the keyword “mosquito vector” in transmission mechanisms is closely linked to the distribution of mosquito vector species and the compatibility of climate zones. The prominence of “zoonosis transmission” in cross-species transmission studies can be analyzed in relation to key nodes within the livestock trade network. Additionally, examining the contribution intensity and timeframe of keywords like “vaccine” and “genetic reassortment” in prevention and control measures can effectively capture the technological breakthroughs in vaccine development, as well as the multidimensional interdisciplinary studies conducted in this area. This analysis may also reflect the correlation between vaccination coverage rates and the scales of outbreaks. In the context of bibliometric analysis intertwined with infectious disease research, the time-slice network analysis map of keywords, along with the period and intensity shown in the keyword outbreak map, can indicate shifts in research focus and track the dynamic pace of epidemic response (23). Moreover, the VOSviewer visualization tool offers insight into collaboration patterns within the research field through a three-dimensional network representing “authors, countries, and institutions.” It also helps identify knowledge gaps via the co-occurrence network map for keywords like “pathogen-host” and “prevention and control measures,” thereby facilitating a comprehensive analysis of the knowledge base and structure within related fields (24).

Prior to the 21st century, RVF was largely endemic to Africa, characterized by a high incidence rate. However, in recent years, there has been a notable increase in extreme weather events worldwide. The transmission dynamics of the RVFV are closely linked to climate change. Heavy rainfall, torrential downpours, and flooding significantly elevate the risk of environmental epidemics affecting both animals and humans. Increased rainfall and higher absolute humidity levels facilitate mosquito reproduction, thus enhancing virus transmission and enabling RVF to expand into previously unaffected regions, such as the Arabian Peninsula (25, 26). Additionally, rising temperatures can improve the survival rates of mosquito vectors, further contributing to the spread of the virus among various susceptible vertebrates (27, 28).

Symptoms of RVF are varied and may include fever, headache, muscle pain, vomiting, diarrhea, and abnormal liver function, which in some cases can lead to liver damage, hemorrhage, neurological issues, and even death (29). In animals, RVF is highly pathogenic, particularly in cattle, sheep, goats, and camels, often resulting in abortions, stillbirths, and significant livestock mortality, leading to severe losses in agricultural production and local economies (30). The diverse and non-specific symptoms of the disease complicate clinical diagnosis and the development of specific therapeutic agents, especially in the early stages. Since its identification in 1931, RVF has posed challenges in differential diagnosis from other viral hemorrhagic fevers and febrile illnesses due to overlapping clinical manifestations. Notably, no licensed human vaccine, specific antiviral therapy, or sufficiently sensitive diagnostic method has been developed to date (31, 32). Most RVF cases are mild and of short duration, not requiring specific treatment, while symptomatic and supportive care remains the mainstay for severe cases. However, some broad-spectrum antiviral drugs, such as Tilorone-dihydrochloride (Tilorone), Galidesivir (BCX4430), and Favipiravir (T-705), have demonstrated potent antiviral effects in mouse models of RVFV infection, showing significant therapeutic efficacy and potential as antiviral therapeutic agents for treating RVFV infections (33–35). Various RVF vaccines (e.g., ChAdOx1 vaccine and MP-12 vaccine) have been tested in animals, but safe and effective vaccines for humans are still in the trial stage (36, 37). Therefore, advancing RVF vaccine development and exploring novel antivirals must remain key research priorities.

Currently, RVF has become one of the important global public health concerns. We integrated bibliometric analysis into the study of RVFV to provide a systematic approach to understanding the evolution, current trends, and future directions of this zoonotic disease. The bibliometric characterization reflects the transformation of the field from initial exploration to gradual maturation in addressing public health challenges. This study primarily utilized literature retrieved from the Scopus database, which was selected for its superior journal coverage and broader availability of abstracts in the natural, life, and health sciences compared to alternative databases such as PubMed and Web of Science (38–42). However, the data sources for this study are primarily limited to English literature available in the Scopus database. While Scopus is an internationally recognized and authoritative academic database, its scope of inclusion does have limitations, meaning that some relevant literature may be overlooked. This selective inclusion could affect the accuracy of citation counts to some extent and may lead to minor deviations in the final analysis results. As technology advances and research continues to evolve, bibliometric visualization analysis techniques are expected to be continually refined, enhancing their accuracy and reliability. This progress will ultimately provide the academic community with more precise and authoritative research support.

Based on the number of articles published in this field, we found that most of the top-ranking institutions are from the United States and France. Consequently, these two countries may hold a prominent academic position in RVF research. Their funding and infrastructure likely provide substantial support for research in this field, and institutions in these countries may engage in a higher level of international collaboration. As a result, regions prone to RVF can enhance their international cooperation with these nations to better address the threat posed by the RVFV in the future. Key strategies to achieve this include strengthening the research capabilities of countries affected by the virus, promoting effective regional collaboration, increasing the involvement of local researchers, and facilitating data sharing among partner countries. Additionally, given the impact of global climate change and international trade, the spread of RVF may further expand. Relevant countries and institutions should raise awareness of prevention and control, optimize the surveillance networks, and advance the research and development of new vaccines and treatments. Through interdisciplinary and multilevel research cooperation, it is anticipated that our understanding of RVF transmission patterns and prevention and control strategies will improve, providing significant scientific support for global public health security.

The analysis presented in this study underscores a notable increase in academic interest correlating directly with global outbreaks of the disease, with significant spikes following major incidents such as the outbreaks in Egypt (1977–1978), the Arabian Peninsula (2000), and South Africa (2010). Current research is primarily concentrated on understanding RVFV pathogenesis, investigating host immune responses, and advancing the development of vaccines and antiviral medications. The leading contributors to RVFV research are identified as the United States and France. We investigate the trajectory of research in the field of RVFV and outline potential future directions through econometric analyses of publication numbers, country distribution, research hotspots, author collaboration networks, and institutional partnerships. This research highlights the urgent need for enhanced global cooperation to elevate the research standards in regions with high disease incidence while underscoring the importance of data sharing and interdisciplinary collaboration. It also notes a shift in focus from basic research to applied strategies for the prevention, control, and treatment of RVFV. Future research on RVF should concentrate on areas where scientific understanding remains incomplete, striving to fill existing knowledge gaps and address potential global health threats. Specifically, the mechanisms underlying strain variation and cross-species transmission require further investigation. The impact of genomic variations of RVFV on virulence, host adaptability, and transmission pathways must be explored in greater depth. Additionally, the existence of undiscovered animal hosts and their roles in the viral ecological cycle remain unknown. Moreover, while extensive research has been conducted on RVF epidemics, the mechanisms enabling the virus to persist during non-epidemic periods are still unclear. The ecological characteristics of potential hosts and vectors, as well as the environmental factors that support the virus’s survival and contribute to epidemic recurrence, have not been fully elucidated. These aspects should be prioritized as core directions for future research. We recommend a multidisciplinary approach that integrates virology, climatology, and public health to effectively combat this global health challenge and provide essential scientific support for controlling the spread of the virus.

## References

[1] Hartman A. Rift Valley Fever. Clin Lab Med. (2017) 37:285–301. doi: 10.1016/j.cll.2017.01.004, PMID: 28457351 PMC5458783

[2] Wright D Kortekaas J Bowden TA Warimwe GM. Rift Valley fever: biology and epidemiology. J Gen Virol. (2019) 100:1187–99. doi: 10.1099/jgv.0.001296, PMID: 31310198 PMC7613496

[3] Kimble JB Noronha L Trujillo JD Mitzel D Richt JA Wilson WC. Rift Valley fever. Vet Clin North Am Food Anim Pract. (2024) 40:293–304. doi: 10.1016/j.cvfa.2024.01.004, PMID: 38453549

[4] Nicholas DE Jacobsen KH Waters NM. Risk factors associated with human Rift Valley fever infection: systematic review and meta-analysis. Trop Med Int Health. (2014) 19:1420–9. doi: 10.1111/tmi.12385, PMID: 25252137

[5] Laughlin LW Meegan JM Strausbaugh LJ Morens DM Watten RH. Epidemic Rift Valley fever in Egypt: observations of the spectrum of human illness. Trans R Soc Trop Med Hyg. (1979) 73:630–3. doi: 10.1016/0035-9203(79)90006-3, PMID: 575446

[6] Smithburn K.C. Mahaffy A.F., and et al. (1949). Rift valley fever; accidental infections among laboratory workers. J Immunol 62, 213–227. 18153372

[7] McElroy AK Nichol ST. Rift Valley fever virus inhibits a pro-inflammatory response in experimentally infected human monocyte derived macrophages and a pro-inflammatory cytokine response may be associated with patient survival during natural infection. Virology. (2012) 422:6–12. doi: 10.1016/j.virol.2011.09.023, PMID: 22018491 PMC6487494

[8] Ikegami T Makino S. The pathogenesis of Rift Valley fever. Viruses. (2011) 3:493–519. doi: 10.3390/v3050493, PMID: 21666766 PMC3111045

[9] Al-Hazmi A Al-Rajhi AA Abboud EB Ayoola EA Al-Hazmi M Saadi R . Ocular complications of Rift Valley fever outbreak in Saudi Arabia. Ophthalmology. (2005) 112:313–8. doi: 10.1016/j.ophtha.2004.09.018, PMID: 15691569

[10] Baudin M Jumaa AM Jomma HJE Karsany MS Bucht G Näslund J . Association of Rift Valley fever virus infection with miscarriage in Sudanese women: a cross-sectional study. Lancet Glob Health. (2016) 4:e864–71. doi: 10.1016/s2214-109x(16)30176-0, PMID: 27692776

[11] Mansfield KL Banyard AC McElhinney L Johnson N Horton DL Hernández-Triana LM . Rift Valley fever virus: a review of diagnosis and vaccination, and implications for emergence in Europe. Vaccine. (2015) 33:5520–31. doi: 10.1016/j.vaccine.2015.08.020, PMID: 26296499

[12] Nair N Osterhaus A Rimmelzwaan GF Prajeeth CK. Rift valley fever virus-infection, pathogenesis and host immune responses. Pathogens. (2023) 12:1174. doi: 10.3390/pathogens12091174, PMID: 37764982 PMC10535968

[13] Clark MHA Warimwe GM Di Nardo A Lyons NA Gubbins S. Systematic literature review of Rift Valley fever virus seroprevalence in livestock, wildlife and humans in Africa from 1968 to 2016. PLoS Negl Trop Dis. (2018) 12:e0006627. doi: 10.1371/journal.pntd.0006627, PMID: 30036382 PMC6072204

[14] van Eck NJ Waltman L. Software survey: VOSviewer, a computer program for bibliometric mapping. Scientometrics. (2010) 84:523–38. doi: 10.1007/s11192-009-0146-3, PMID: 20585380 PMC2883932

[15] LaBeaud AD Kazura JW King CH. Advances in Rift Valley fever research: insights for disease prevention. Curr Opin Infect Dis. (2010) 23:403–8. doi: 10.1097/QCO.0b013e32833c3da6, PMID: 20613512 PMC3126654

[16] Campbell LP Alexander AM. Landscape genetics of Aedes mcintoshi (Diptera: Culicidae), an important vector of Rift Valley fever virus in northeastern Kenya. J Med Entomol. (2017) 54:1258–65. doi: 10.1093/jme/tjx072, PMID: 28431166

[17] Johnson BK Chanas AC el-Tayeb E Abdel-Wahab KS Sheheta FA Mohamed Ae-D. Rift Valley fever in Egypt, 1978. Lancet. (1978) 2:745. doi: 10.1016/s0140-6736(78)92753-8 80678

[18] Mhamadi M Badji A Barry MA Ndiaye EH Gaye A Ndiaye M . Human and livestock surveillance revealed the circulation of Rift Valley fever virus in Agnam, northern Senegal, 2021. Trop Med Infect Dis. (2023) 8:87. doi: 10.3390/tropicalmed8020087, PMID: 36828503 PMC9962223

[19] Jost CC Nzietchueng S Kihu S Bett B Njogu G Swai ES . Epidemiological assessment of the Rift Valley fever outbreak in Kenya and Tanzania in 2006 and 2007. Am J Trop Med Hyg. (2010) 83:65–72. doi: 10.4269/ajtmh.2010.09-0290, PMID: 20682908 PMC2913500

[20] CDC. Outbreak of rift valley fever--Yemen, august-October 2000. MMWR Morb Mortal Wkly Rep. (2000) 49:1065–6. 11186611

[21] Métras R Porphyre T Pfeiffer DU Kemp A Thompson PN Collins LM . Exploratory space-time analyses of Rift Valley fever in South Africa in 2008-2011. PLoS Negl Trop Dis. (2012) 6:e1808. doi: 10.1371/journal.pntd.0001808, PMID: 22953020 PMC3429380

[22] Rostal MK Cleaveland S Cordel C Staden LV Matthews L Anyamba A . Farm-level risk factors of increased abortion and mortality in domestic ruminants during the 2010 Rift Valley fever outbreak in Central South Africa. Pathogens. (2020) 9:914. doi: 10.3390/pathogens9110914, PMID: 33158214 PMC7694248

[23] Zhang H Zhang L. Knowledge mapping of severe fever with thrombocytopenia syndrome: a bibliometric analysis. Front Microbiol. (2024) 15:1423181. doi: 10.3389/fmicb.2024.1423181, PMID: 39139373 PMC11319145

[24] Zhang H Zhang L. A bibliometric and visualized analysis of heartland virus. Front Microbiol. (2024) 15:1509749. doi: 10.3389/fmicb.2024.1509749 39872819 PMC11770020

[25] Chemison A Ramstein G Jones A Morse A Caminade C. Ability of a dynamical climate sensitive disease model to reproduce historical Rift Valley fever outbreaks over Africa. Sci Rep. (2024) 14:3904. doi: 10.1038/s41598-024-53774-x, PMID: 38365824 PMC10873308

[26] Rahman MM Islam MR Dhar PS. Recent re-emergence of Rift Valley fever: epidemiology, clinical characteristics, transmission, symptoms, diagnosis, prevention, and treatment. Int J Surg. (2023) 109:117–9. doi: 10.1097/js9.0000000000000043, PMID: 36799821 PMC10389314

[27] Linthicum KJ Britch SC Anyamba A. Rift Valley fever: an emerging mosquito-borne disease. Annu Rev Entomol. (2016) 61:395–415. doi: 10.1146/annurev-ento-010715-023819, PMID: 26982443

[28] Paweska JT. Rift Valley fever. Rev Sci Tech. (2015) 34:375–89. doi: 10.20506/rst.34.2.2364, PMID: 26601442

[29] Kitandwe PK McKay PF Kaleebu P Shattock RJ. An overview of Rift Valley fever vaccine development strategies. Vaccines (Basel). (2022) 10:1794. doi: 10.3390/vaccines10111794, PMID: 36366303 PMC9697312

[30] Ahmed A Ali Y Elduma A Eldigail MH Mhmoud RA Mohamed NS . Unique outbreak of Rift Valley fever in Sudan, 2019. Emerg Infect Dis. (2020) 26:3030–3. doi: 10.3201/eid2612.201599, PMID: 33219787 PMC7706939

[31] Lapa D Pauciullo S Ricci I Garbuglia AR Maggi F Scicluna MT . Rift Valley fever virus: an overview of the current status of diagnostics. Biomedicine. (2024) 12:540. doi: 10.3390/biomedicines12030540, PMID: 38540153 PMC10968371

[32] Wu W Zhang S Qu J Zhang Q Li C Li J . Simultaneous detection of IgG antibodies associated with viral hemorrhagic fever by a multiplexed Luminex-based immunoassay. Virus Res. (2014) 187:84–90. doi: 10.1016/j.virusres.2013.12.037, PMID: 24631566

[33] Caroline AL Powell DS Bethel LM Oury TD Reed DS Hartman AL. Broad spectrum antiviral activity of favipiravir (T-705): protection from highly lethal inhalational Rift Valley fever. PLoS Negl Trop Dis. (2014) 8:e2790. doi: 10.1371/journal.pntd.0002790, PMID: 24722586 PMC3983105

[34] Johnson KN Kalveram B Smith JK Zhang L Juelich T Atkins C . Tilorone-Dihydrochloride protects against Rift Valley fever virus infection and disease in the mouse model. Microorganisms. (2021) 10:92. doi: 10.3390/microorganisms10010092, PMID: 35056541 PMC8781158

[35] Westover JB Mathis A Taylor R Wandersee L Bailey KW Sefing EJ . Galidesivir limits Rift Valley fever virus infection and disease in Syrian golden hamsters. Antivir Res. (2018) 156:38–45. doi: 10.1016/j.antiviral.2018.05.013, PMID: 29864447 PMC6035881

[36] Bronder S Sester M. A novel Rift Valley fever vaccine. Lancet Infect Dis. (2023) 23:887–9. doi: 10.1016/s1473-3099(23)00134-2, PMID: 37060918

[37] Saluzzo JF Smith JF. Use of reassortant viruses to map attenuating and temperature-sensitive mutations of the Rift Valley fever virus MP-12 vaccine. Vaccine. (1990) 8:369–75. doi: 10.1016/0264-410x(90)90096-5, PMID: 2396475

[38] Chen R Wang Z Zhang L. Research trends on alphavirus receptors: a bibliometric analysis. Front Cell Infect Microbiol. (2024) 14:1388360. doi: 10.3389/fcimb.2024.1388360, PMID: 38841111 PMC11150648

[39] Dong J Li Z Gao S Zhang L. A bibliometric analysis of Oropouche virus. Front Microbiol. (2024) 15:1457773. doi: 10.3389/fmicb.2024.1457773, PMID: 39444684 PMC11496263

[40] Martín-Martín A Thelwall M Orduna-Malea E Delgado López-Cózar E. Google scholar, Microsoft academic, Scopus, dimensions, web of science, and OpenCitations' COCI: a multidisciplinary comparison of coverage via citations. Scientometrics. (2021) 126:871–906. doi: 10.1007/s11192-020-03690-4, PMID: 32981987 PMC7505221

[41] Powell KR Peterson SR. Coverage and quality: a comparison of web of science and Scopus databases for reporting faculty nursing publication metrics. Nurs Outlook. (2017) 65:572–8. doi: 10.1016/j.outlook.2017.03.004, PMID: 28377037

[42] Wang Y Wang Z Cui H Zhang L. The migrasome as a developmental learning paradigm in cell biology. Cell Biol Int. (2024) 48:1254–65. doi: 10.1002/cbin.12220, PMID: 39010645

